# CIS-based registration of quality of life in a single source approach

**DOI:** 10.1186/1472-6947-11-26

**Published:** 2011-04-21

**Authors:** Fleur Fritz, Sonja Ständer, Bernhard Breil, Markus Riek, Martin Dugas

**Affiliations:** 1 Institute of Medical Informatics, University Münster, Domagkstraße 9, 48149 Münster, Germany; 2 Department of Dermatology, Competence Center for the Diagnosis and Therapy of Chronic Pruritus, University Hospital Muenster, Münster, Germany

**Keywords:** Quality of Life, Pruritus, Single Source, Clinical Information System, Medical Documentation, Mobile Device, Web-based Application, Patient Questionnaire, Data Import

## Abstract

**Background:**

Documenting quality of life (QoL) in routine medical care and using it both for treatment and for clinical research is not common, although such information is absolutely valuable for physicians and patients alike. We therefore aimed at developing an efficient method to integrate quality of life information into the clinical information system (CIS) and thus make it available for clinical care and secondary use.

**Methods:**

We piloted our method in three different medical departments, using five different QoL questionnaires. In this setting we used structured interviews and onsite observations to perform workflow and form analyses. The forms and pertinent data reports were implemented using the integrated tools of the local CIS. A web-based application for mobile devices was developed based on XML schemata to facilitate data import into the CIS. Data exports of the CIS were analysed with statistical software to perform an analysis of data quality.

**Results:**

The quality of life questionnaires are now regularly documented by patients and physicians. The resulting data is available in the Electronic Health Record (EHR) and can be used for treatment purposes and communication as well as research functionalities. The completion of questionnaires by the patients themselves using a mobile device (iPad) and the import of the respective data into the CIS forms were successfully tested in a pilot installation. The quality of data is rendered high by the use of automatic score calculations as well as the automatic creation of forms for follow-up documentation. The QoL data was exported to research databases for use in scientific analysis.

**Conclusion:**

The CIS-based QoL is technically feasible, clinically accepted and provides an excellent quality of data for medical treatment and clinical research. Our approach with a commercial CIS and the web-based application is transferable to other sites.

## Background

The patient's quality of life (QoL) data is a valuable tool for measuring the wellbeing of a patient following medical treatment [1] and may be used as a parameter for both routine care [2] and clinical research in the respective medical field [3,4]. QoL data can also be used in prognostic factor analysis [5]. Asking the patient about QoL provides information which is unavailable from other sources [6]. The discrepancy between the patient's view and the clinical view on illness and treatment becomes transparent through the use of QoL data and enables the physician to obtain a better understanding of the patient. This may lead to an improved quality of care and encourage patient compliance [7,8]. Furthermore with regard to health economic questions such information is relevant to argue for the refund of a special treatment or more recent and expensive products by third party payers. Finally the pharmaceutical industry and manufacturers of medicinal products have a large interest in assessing the views of the patients on product and clinical efficacy to enhance their research and achieve a competitive advantage [7].

QoL is normally documented by using paper-based questionnaires, to be filled in either directly by the patients or by their treating physicians. In the latter case it is not a self-assessment. In both cases, however, the questionnaires are patient related and will therefore uniformly be referred to as patient questionnaires throughout this article. The completed questionnaire usually results in one or more scores (for different categories within the form) describing the impairment of QoL. This impairment might be related to overall health, to functional disorders or to specific diseases [9]. The QoL questionnaires are mainly classified into these categories and into those of the overall concept of health, relating to the respective physical, psychological, and social aspects [10,11]. Wilson and Cleary proposed a classification scheme based on five levels: biological and physiological factors, symptoms, functioning, general health perceptions and overall quality of life. They also state that traditional clinical variables are linked to those health related quality of life measures and emphasize the relevance of their systematic application to improve patient outcomes [12].

QoL today is still rarely documented in routine healthcare [13] although it was shown that the systematic assessment is beneficial for medical staff as well as patients, and the latter can and will complete almost any form regarding their outcomes [7,8,14]. Physicians spend approximately 25% of their working time with routine documentation tasks [15], which might explain why they do not take the time to systematically register extra information not urgently needed. Testa and Simonson have already stated in 1996 that the assessment of patient reported outcomes will need to "demonstrate the links among medical interventions, clinical and physiologic changes, and the quality of life so that the practicing physician can better understand the clinical implications of these measures" [9]. There is also a lack of studies in clinical research which take QoL data into account [16] although in pharmaceutical Phase III studies their use has grown or has even become mandatory [7]. Some clinical researchers collect this kind of data for retrospective studies. However, in the context of research such information is for the most part collected separately and stored outside the CIS, impending the promotion of such research amongst physicians [17].

The separate collection of clinical and research data implies that the same data is processed in duplicate, which leads to the use of additional time and resources. Different sources also lead to error-prone data when it is reused and transferred into a single database [18]. The main goal of a single source approach is to combine clinical routine and research documentation and consequently use only one source of information [19]. Thus time for redundant documentation can be saved and more routine data, even if collected for different purposes [20], are available for research questions. If information about QoL were documented during clinical routine by the patients themselves and transferred to the CIS, it would not add documentation time to the physician's workload and the available data could be used in multiple ways. For example:

- Physicians could refer to the impairment on QoL during an interview with the patient and consider it for their choice and subsequent evaluation of treatment.

- Information about QoL could be transferred to the primary care physician by integrating it in the discharge letter.

- New treatment methods could be justified to third party payers.

- For long-term treatments the effects might be analysed using progress data about QoL.

- Together with other routine clinical data like diagnosis, treatment, laboratory values and the course of disease QoL could serve as an additional parameter for various clinical studies as well as for quality management purposes.

As QoL is a key element in the clinical assessment a solution is needed to overcome the obstacles of its documentation in current care and involve the patients directly. Our objectives are therefore to develop an efficient method to document QoL during the clinical practice, make the documented data available within the CIS and use it both for clinical routine and research.

## Methods

### Setting

We have been working with three departments at the University Hospital of Muenster covering different disease areas to assess the general concept, which was identical for all three departments.

The first area, which for this article is the primary object, is the Competence Center for the Diagnosis and Therapy of chronic Pruritus within the Department of Dermatology (short: pruritus center). The pruritus center offers treatment for more than 1700 patients a year and has already gained experience in documenting QoL. Moreover, extensive research is being done in this field as pruritus dramatically affects the patients' quality of life [21,22]. As a tool to document the QoL in dermatology the Dermatology Life Quality Index (DLQI) by A. Finley was chosen as a widespread and well accepted score [23,24].

The second area is the Department of Psychiatry, treating approximately 450 inpatients with mood disorders a year. Amongst others data about these disorders has been collected since 2004 for a long term clinical study in which an average of 15 patients is enrolled per month. Three different questionnaires are used for scoring: The Beck Depression Inventory (BDI), which is filled in by the patient on admission and discharge [25], the Hamilton Rating Scale for Depression (HAMD) [26] and the Young Mania Rating Scale (YMRS), which are filled in on a weekly basis by the treating physician [27].

The third area is oncology where patients are encouraged to a consultation of the Department of Psychosomatic Medicine, if they have a high anxiety and depression score. This score is documented using the Hospital Anxiety and Depression Scale (HADS) [28], which is currently filled in by 250 patients per month (on average); a quarter of them show a critical score. This score is also being used by other departments such as the pruritus center, which uses the HADS for all their patients.

Although the questionnaires in psychiatry and oncology are not explicitly QoL scores, they are directly correlated with such scores and reflect how the disease affects the patient's life. While the aim and content of different questionnaire types is surely to be taken into consideration [11], the requirements of informatics and the general structure of the forms are comparable for all three domains and all analysed questionnaires. We therefore use the term QoL questionnaires for all of them in our concept.

The project was implemented in two stages. First the paper-based forms were implemented within the CIS that is used by the medical staff. During that stage the questionnaires completed by the patients had to be transferred manually into the CIS. In a second stage a web-based application was developed to enable the electronic documentation of the questionnaires by the patients themselves.

### Workflow and form analysis

The different workflows and the respective documentation were analysed using standard methods of business workflow analysis. The workflows were analysed before and after form implementation in the CIS. Structured interviews, onsite observation and form analysis were conducted [29]. Workflows were illustrated with basic flowcharts by Microsoft Visio [30].

### Implementation of forms within the clinical information system

According to the official guidelines from the DLQI questionnaire, the BDI, the HAMD, the YMRS and the HADS all forms were parameterised using the integrated tool (ORBIS^® ^Composer) of the local clinical information system ORBIS^® ^by Agfa Healthcare [31]. To include QoL into the letters of a physician a text module was created which transfers the score of the form into a text phrase. A link to an overview form, that collects and displays all previously entered data, was embedded. For disease areas where a questionnaire has to be filled in regularly, a system was installed which automatically creates a form every week until discharge, following the patient's admittance and the first manual creation of a form [32].

### Implementation of patient reported questionnaires in a web-based application

The questionnaires in the web-based application are based on an XML schema [33], which can be found in additional file 1 in the supplement. According to this schema, content and metadata from the questionnaires can be exported into an XML format. To import this file into various CIS, a mapping to the respective form structure can be defined through the web-based application [34]. The mapping to our local CIS was defined and an XML import function was implemented in the CIS form. A designated folder was created in the file system for the XML import files which is used by the web-based application and the local CIS.

### Reporting functionality

The local clinical information system's integrated tool (ORBIS^® ^Report Designer [31]) was used to extract the data from the CIS for further purposes. Reports were embedded in the overview form to query previously entered data. Pseudonymised exports of queried data were generated as comma separated value (csv) files. Descriptive statistics, based on the extracted data, were subsequently calculated using SPSS Statistics [35].

### Data quality analysis

The data quality analysis method which was used involved querying the CIS to calculate numbers related to documented questionnaires, patient status, completeness of forms (defined as the number of documented forms compared to the number of patient appointments) and reuse of data within other forms.

### Statement of ethics

The study was performed in compliance with the World Medical Association Declaration of Helsinki on Ethical Principles for Medical Research Involving Human Subjects. Patients gave their informed consent before participating in the questionnaires. In addition local German regulations allow the use of clinical data collected in a university hospital setting for research purposes. Access to the CIS data was authorized by the treating physician and the director of the clinic and all extracted CIS data was pseudonymised. The whole technical concept was approved by the responsible data protection officer of the University Hospital in Muenster.

## Results

Three different departments with their respective QoL documentation were analysed. The form analysis showed a similar structure for all questionnaires: There are 10 - 21 questions (DLQI = 10, HADS = 14, YMRS = 11, HAMD = 21, BDI = 21) that can be answered via radio buttons with three to five values representing defined numerical values. According to specific guidelines for each questionnaire a score can be calculated from the values. This can either be done for the sum of all questions or for subsets which represent different categories of QoL, for example anxiety and depression in the HADS. Usually a minimum number of questions have to be answered in order to calculate a valid score (e.g. one missing value for each category is allowed in the HADS).

In the following we will show the generalised process for all three and exemplarily provide more specific data for the DLQI used in the pruritus center.

The official instructions of the questionnaires facilitated their parameterisation into the local CIS and an average of one workday per form was needed for design and implementation. In the first stage of the project the questionnaires were solely used in the CIS and the patient-based questionnaires had to be transferred manually. To have a full electronic workflow, we then developed the web-based application for mobile devices and piloted it in one department. This application was designed and implemented during a 2-month bachelor thesis. The numbers about usage in this paper are mainly based on the data received in the first stage of the project as the pilot phase of the second stage did not last long enough to receive a sufficient amount of data.

### Previous and new documentation workflow

The patient questionnaire workflow before implementation in the CIS was as follows:

The patient receives a paper form of the questionnaire at the beginning of each visit (first contact and follow-up). This is filled in while the patient awaits his/her consultation. When completed, the patient hands the form either to the consulting physician or to the administrative medical staff. The score is then calculated manually and the paper sheet is filed away in the medical record. The resulting information can be used for treatment purposes. If the score is needed for scientific purposes, it can be entered into a separate research database by hand. These patient questionnaires have not been used regularly and in most cases only for single research projects.

**Figure 1:**
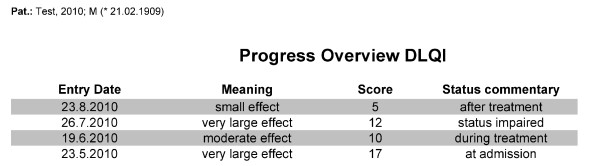
**Progress overview for one patient with entry date, commentary on the status when DLQI was documented, the DLQI score for that date and the respective meaning of the score**.

In the first stage of the project these forms were implemented in the local CIS and the medical staff received on-the-job-training. The training duration was 15 minutes and covered all aspects related to accessing and using the forms available in the CIS. One to two users were trained and these users then trained their colleagues when needed. We implemented patient questionnaires as well as accompanying forms, such as a cumulative report to provide a score overview for an individual patient as shown in figure 1.

The paper-based forms received from the patients were manually transferred into the CIS forms by medical and administrative staff respectively.

In the second stage of the project we added the web-based application, which allows the patients to complete an electronic version of the questionnaires on a mobile device, in this case using an iPad [36]. Upon implementation of the mobile device the medical staff no longer needed to manually transfer the data.

The workflow after the complete implementation is as follows:

In the case of a patient's self-assessment upon his/her arrival, the administrative medical staff enters the patient's case ID into the web-based application using a mobile device (iPad) and chooses the respective questionnaire. Once the questionnaire is loaded, user rights adapt and the device is handed to the patient. The patient fills in the questionnaire while awaiting his/her consultation. After completion the patient hands the mobile device to the administrative medical staff. By pressing an import button in the respective form within the CIS, the form data is imported by means of an XML file. In the equivalent form in the CIS the score is calculated automatically and gets stored in the patient's electronic health record (EHR). In case the treating physician has to complete the questionnaire he/she can document the respective form directly in the EHR. From there it can be used:

- for purposes of treatment including an overview of all previously documented scores available within each form,

- for purposes of communication, e.g. transfer into the physician's letter and

- to report functionalities for quality management and research projects.

For the latter the QoL data can be exported into a csv file suitable for standard statistics software.

**Figure 2:**
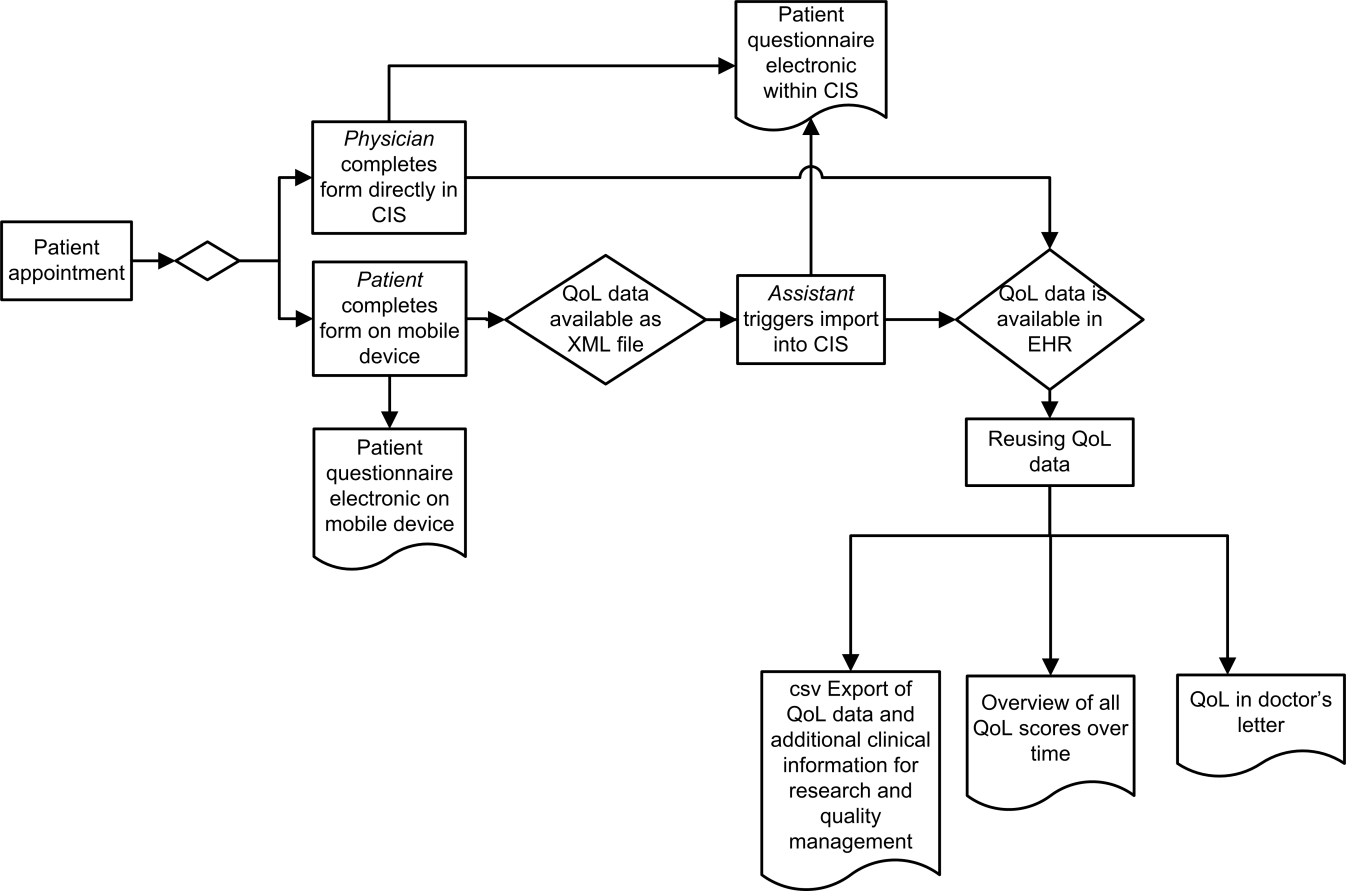
**Workflow of QoL documentation after the implementation of the form on a web-based application and in the CIS**: The patient arrives at his/her appointment and receives a mobile device to complete a patient questionnaire. The XML file is imported into the CIS form by an assistant. In case it is not a self-assessment but a physician-based questionnaire, the treating physician will document the form directly in the CIS. In both cases the data about QoL are now available in the patient's EHR and can be reused for routine treatment and clinical research.

Figure 2 shows the workflow of QoL documentation after the forms were implemented in the CIS.

### Efficient documentation and data quality

**Figure 3:**
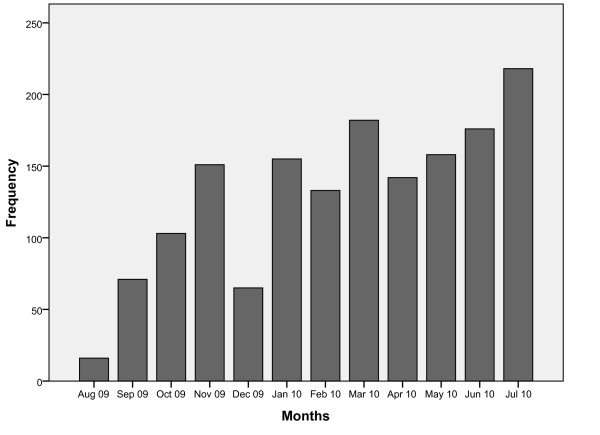
**Number of DLQI questionnaires since implementation in August 2009**. Apart from a decrease around Christmas/year-end, the figure shows an increase in the use of DLQI for patients.

As can be seen in figure 3, 1600 DLQI questionnaires were entered into the CIS in the pruritus center in the course of one year. 84% of them were filled in for outpatients, 16% for inpatients. The number of forms includes follow-up forms being filled in for 900 patients and 1400 medical cases respectively. The use of the DLQI did not decrease after the introduction phase; it increased to 220 questionnaires in one month.

For a six-month period (01 February 2010 until 01 August 2010) we compared the number of patients having an outpatient appointment at the pruritus center (extracted out of the CIS calendars) with the number of completed DLQI forms. During 1122 outpatient appointments, 880 DLQI forms were filled in which results in 78% completeness of forms.

The DLQI score was transferred into the physician's letter for 30% of all inpatients.

As the web-based application includes rules for the score calculation (according to the respective questionnaire guidelines), only complete forms will generate an XML file to be imported into the CIS. For the forms which are directly documented within the CIS, the score will only be displayed if complete. In this case, for reporting functionalities, missing values can be handled separately. For the above mentioned 880 DLQI forms, including those which were entered directly into the CIS, the report shows 800 forms (91%) having a score and thus being filled in completely and 80 forms (9%) without a score because of missing item values.

To maximize form completeness, we used a system that automatically creates the necessary forms on a weekly basis for two types of questionnaires in psychiatry. These questionnaires are filled in by the physician instead of the patient himself (HAMD and YMRS). This system is triggered once a form is created manually for a patient and stops when the patient is discharged. These forms appear in a work list from which the medical staff can complete and save the questionnaires for the respective patients. In a three-month period (10 May 2010 until 10 August 2010) 92% of all inpatients had weekly documentation.

### Reporting functionality - Use in routine care and in clinical research

From the questionnaires all data items, the resulting score, the meaning of the score and the administrative data (date of form creation, study/patient ID, age and gender) can be exported in csv format. Other relevant clinical data available in the CIS can also be added such as the progress of symptoms, diagnosis, clinical procedures, medical history data and clinical notes. In the case of clinical studies a study number can be entered into the form and pseudonymised data related to the study can be exported.

**Figure 4:**
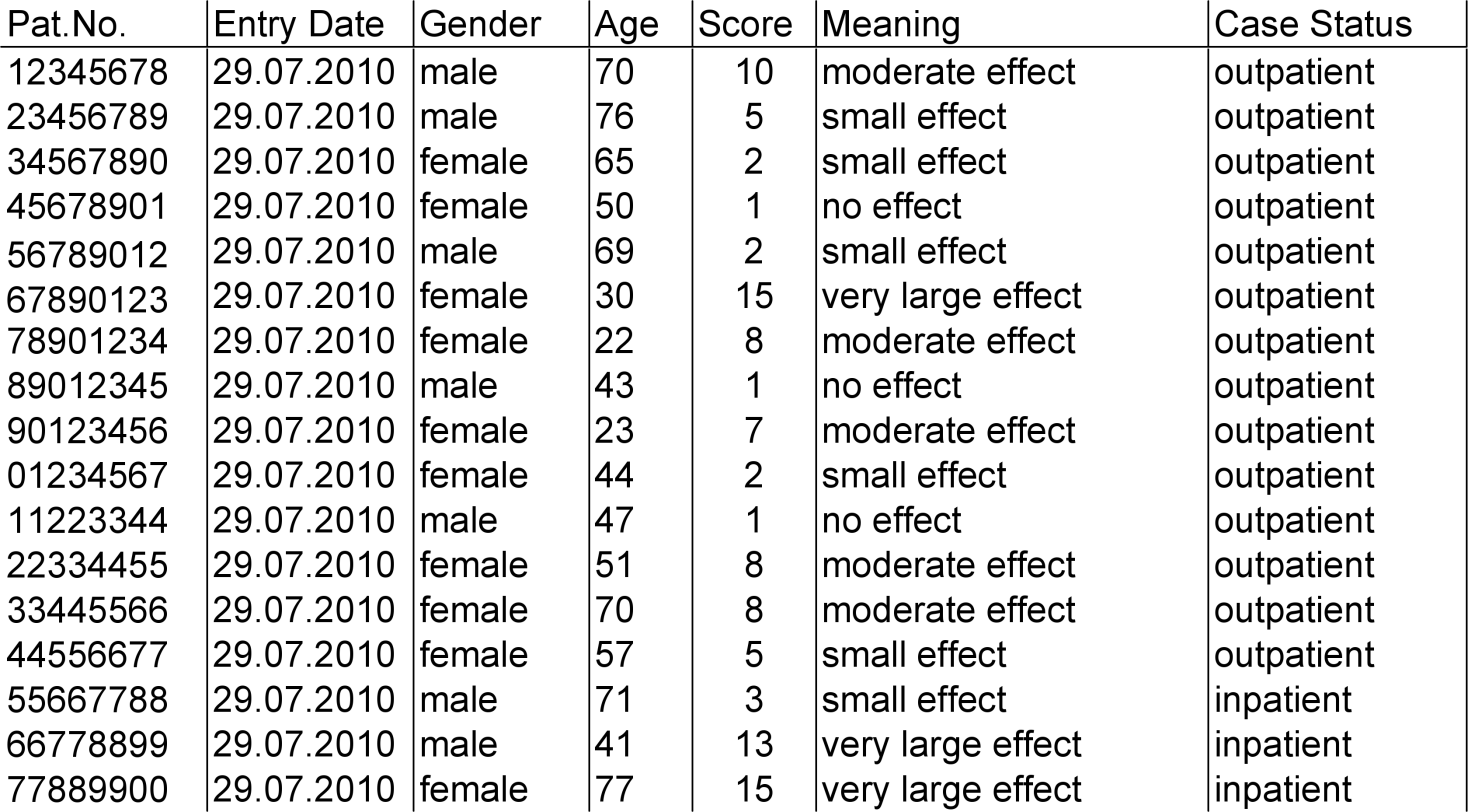
**Extract of a data export of the DLQI form with pseudonymised patient number, medical entry date of the form, gender of the patient, age of the patient, the resulting DLQI score, the meaning of the score, and the case status of the patient**.

Figure 4 shows an example of data export of the DLQI form.

## Discussion

### Integration into clinical practice

To overcome the obstacles of registering QoL in the clinical practice, we implemented a solution that involves the patients and incorporates their respective data in the EHR. The technical solution was successfully tested and, because physicians noticed that little or no additional time is needed when patients complete the questionnaires while awaiting their consultation, our solution was integrated in everyday practice. The discussion about the usage numbers below gives measurable evidence of this. Having QoL data available within the EHR makes it possible to view the progress of QoL for single patients during their treatment, to transfer this data via the physician's letter to the primary care physician and to incorporate this information in the continuity of care. To plan and evaluate the treatment, QoL is taken into consideration as an additional parameter and adds value to the clinical practice [7,8]. Furthermore, it can serve as a justification for new treatments reimbursed by third party payers. For research questions the QoL data can be combined with relevant clinical data available in the EHR. The resulting data exports can be imported in already existing research databases. For instance, in psychiatry the data is transferred into an existing SPSS database [37].

### Data quality and usage

To reach valid conclusions in research and employ CIS data for quality management, it is crucial to have high data quality. The percentage of completed DLQI forms compared to the number of outpatient appointments in the pruritus center shows that high completeness of forms was reached. According to the responsible physicians the missing forms (22% relative to all patient appointments) were due to unwillingness or incapability (mainly due to old age or language problems) to participate in answering questions about QoL in general. The high number of forms also shows good clinical user acceptance as the questionnaires were incorporated into their clinical practice. The DLQI became a relevant parameter in the routine documentation, whereas until then it was only collected intermittently for single research questions in defined periods of time. As already shown by Doward et al., Basch and Salek et al. this is a major benefit for both patient and clinician [7,8,14].

Due to the automatic calculation of the score there no longer are wrong values. The system will only calculate the score if the required number of questions has been answered and only complete forms are exported for research questions. However, forms without a score can also be queried in order to be completed. Using the web-based application there are no more missing values as the system indicates unanswered questions to the user and only completed questionnaires are imported into the CIS.

If regular scores are needed, as is the case for the HAMD and YMRS in psychiatry, the automatic weekly creation of the forms and work lists of patients with incomplete forms support the achievement of a high completeness rate of forms. It might even be possible to use a reminder system that will send emails to the respective medical staff if a form is due. This system has proved its effectiveness in previous studies [32].

### Technical implementation

Our approach was implemented in a commercial CIS and is therefore transferable to all other customers using the same CIS. As the tools for generating forms and reports are available with the same or similar functionality in other commercial CIS, the concept should be usable in those contexts as well, which has yet to be assessed. It can however be said that our approach is not focused on proprietary institution- and provider-centred applications [38] but provides a general concept. The time needed for designing and implementing the forms in the CIS and the web-based application was acceptable, and from our perspective cannot be seen as an obstacle. However, setup and maintenance of the application and the CIS interface requires additional resources.

To encourage other hospitals to implement patient questionnaires and define standards enabling the possibility of comparing data, we provided a general XML schema which describes the structure and content of the forms (see additional file 1). According to this schema specialized questionnaires can be defined. The resulting XML files can be mapped with respect to the different variable names coming from different CIS using the mapping functionality of the web-based application. Our experience has shown that such an implementation required one workday per CIS form.

We chose a web-based application to allow the use of questionnaires with various devices; however, a specialized application for the iPad might be a future add-on.

### Other effects

Using patient questionnaires also aims at increasing the quality of clinical treatment. In the case of the HADS, which is often documented for oncology patients, we also implemented a function to facilitate decision support. If the scores for anxiety or depression reach a critical value, a highlighted link will appear enabling the treating physician to directly request a psychosomatic consultation for the respective patient, resulting in an enhanced quality of care. The usefulness and impact of this support mechanism will have to be evaluated in a future project.

The QoL data of treated patients and the data quality information, such as the completeness of forms, is also interesting for quality management assessments of the respective medical departments. For example, for our local breast cancer center it was required to implement the HADS in order to apply for third party certification. The Department of Psychosomatic Medicine can evaluate their capacity required for patients who need consultation because of critical scores. Furthermore, the medical staff of the pruritus center can assess their goal to reach full completeness of QoL forms for their patients.

### Limitations and future prospects

During informal interviews after the implementation, the main users reported a high overall satisfaction with the electronic version of the questionnaires and favoured them over paper based forms. Nevertheless, a comprehensive evaluation study with appropriate measurement indicators is needed. We will analyse our system in particular with respect to usability and user acceptance both by the medical staff and by the patients. Especially patients of old age might find it difficult to use a mobile device instead of pen and paper. During our first pilot installation some patients showed resistance to use the touchpad, they pressed too hard or too softly. Consequently we will evaluate the use of a touch pen. The medical staff have not shown any resistance so far, but in the pilot setting most users were open-minded with respect to the use of IT. In a full evaluation setting we will integrate more types of users.

Due to the current manual import mechanism the QoL data is only electronically available to the treating physician once the assistant has opened the form in the CIS and imported the XML file. To optimize this process an automatic mechanism to import the XML files into the respective forms within the CIS will be implemented.

The information about the QoL of patients is especially valuable if it can be combined with other clinical data, as for most research questions it is only used as an additional parameter. Therefore our future aim is to include more relevant data in the electronic documentation. For one long term research project in psychiatry this has already been done and in addition to HAMD, YMRS and BDI forms to capture admission and discharge data, for example, about diagnosis, medication and therapy were implemented in the CIS. The whole dataset can be exported into a separate research database [37]. In the dermatology department we currently analyse and consequently describe a pruritus data model regarding the complete documentation, including QoL data in order to enable multicentre studies and use comprehensive data for clinical research.

After having integrated QoL data in clinical treatment, another future research topic will be to analyse the impact of the availability of QoL information on the treatment. Suitable measures will have to be defined and monitored over a longer period of time in order to detect any association.

## Conclusion

The QoL data is now documented in a standard CIS including mobile devices for patient-based questionnaires. The resulting data can be used in routine medical care and can be exported for research purposes. Thus the collection of QoL information with a high quality of data, by using a single source approach, is technically feasible, clinically well accepted and transferable to other sites.

## Abbreviations

BDI: Beck Depression Inventory; CIS: Clinical Information System; CSV: Comma Separated Value; DLQI: Dermatology Life Quality Index; EHR: Electronic Health Record; HADS: Hospital Anxiety and Depression Scale; HAMD: Hamilton Rating Scale for Depression; QoL: Quality of Life; YMRS: Young Mania Rating Scale.

## Competing interests

The authors declare that they have no competing interests.

## Authors' contributions

FF analysed the workflow, implemented the CIS forms and reports, analysed data quality and user acceptance and wrote the manuscript. MR and BB developed the web-based application. SS provided clinical data and the first pilot setting. SS, BB and MD critically revised the manuscript. All authors have read and approved the final manuscript.

## Pre-publication history

The pre-publication history for this paper can be accessed here:

http://www.biomedcentral.com/1472-6947/11/26/prepub

## Supplementary Material

Additional file 1 **This file shows the XML schema for our patient questionnaires**. Click here for file
