# Supplementary material for: CIS-based registration of quality of life in a single source approach
Source: BMC Med Inform Decis Mak. 2011 Apr 21;11:26. doi: 10.1186/1472-6947-11-26 (PMC3107772; doi:10.1186/1472-6947-11-26)
Supplement: Additional file 1 — This file shows the XML schema for our patient questionnaires. [file 1472-6947-11-26-S1.PDF]

```
  <?xml version="1.0" encoding="UTF-8" ?>
- <xs:schema xmlns:xs="http://www.w3.org/2001/XMLSchema" elementFormDefault="qualified">
    <!-- Datatypes - Questionnaire metadata -->
  - <xs:simpleType name="clinic_id">
    - <xs:restriction base="xs:string">
        <xs:minLength value="3" />
        <xs:maxLength value="6" />
      </xs:restriction>
    </xs:simpleType>
  - <xs:complexType name="patient">
    - <xs:sequence>
        <xs:element name="patient_id" type="patient_type" />
        <xs:element name="name" type="xs:string" minOccurs="0" maxOccurs="1" />
        <xs:element name="firstname" type="xs:string" minOccurs="0" maxOccurs="1" />
        <xs:element name="birthdate" type="xs:date" minOccurs="0" maxOccurs="1" />
        <xs:element name="female" type="xs:boolean" minOccurs="0" maxOccurs="1" />
      </xs:sequence>
    </xs:complexType>
  - <xs:simpleType name="patient_type">
    - <xs:restriction base="xs:integer">
        <xs:pattern value="[0-9]{10}" />
      </xs:restriction>
    </xs:simpleType>
    <!-- Datatypes - Questionnaire -->
  - <xs:complexType name="category">
    - <xs:sequence>
        <xs:element name="category_id" type="xs:positiveInteger" />
        <xs:element name="name" type="xs:string" />
        <xs:element name="minimum_answers" type="xs:positiveInteger" />
        <xs:element name="score" type="xs:integer" minOccurs="0" maxOccurs="1" />
      </xs:sequence>
    </xs:complexType>
  - <xs:simpleType name="answer_text">
    - <xs:restriction base="xs:string">
        <xs:minLength value="2" />
        <xs:maxLength value="255" />
      </xs:restriction>
    </xs:simpleType>
  - <xs:simpleType name="answer_value">
    - <xs:restriction base="xs:integer">
        <xs:minInclusive value="0" />
        <xs:maxInclusive value="10" />
      </xs:restriction>
    </xs:simpleType>
  - <xs:complexType name="answer">
    - <xs:sequence>
        <xs:element name="answer_id" type="xs:positiveInteger" />
        <xs:element name="choosed" type="xs:boolean" default="false" />
        <xs:element name="text" type="answer_text" />
        <xs:element name="value" type="answer_value" />
      </xs:sequence>
    </xs:complexType>
    <!-- Questionnaire -->
  - <xs:element name="questionnaire">
    - <xs:complexType>
      - <xs:sequence>
          <!-- Questionnaire metadata -->
        - <xs:element name="head">
          - <xs:complexType>
            - <xs:sequence>
                <xs:element name="questionnaire_id" type="xs:positiveInteger" />
                <xs:element name="name" type="xs:string" minOccurs="0" maxOccurs="1" />
                <xs:element name="description" type="xs:string" minOccurs="0" maxOccurs="1" />
                <xs:element name="comment" type="xs:string" minOccurs="0" maxOccurs="1" />
                <xs:element name="score" type="xs:integer" minOccurs="0" maxOccurs="1" />
                <xs:element name="clinic" type="clinic_id" />
                <xs:element name="patient" type="patient" />
                <xs:element name="timestamp" type="xs:dateTime" />
              - <xs:element name="categories">
                - <xs:complexType>
                  - <xs:sequence>
                      <xs:element name="category" type="category" maxOccurs="10" />
                    </xs:sequence>
                  </xs:complexType>
                </xs:element>
              </xs:sequence>
            </xs:complexType>
          </xs:element>
          <!-- Questions -->
```

```
    - <xs:element name="body">
      - <xs:complexType>
        - <xs:sequence>
          - <xs:element name="chapter" maxOccurs="10">
            - <xs:complexType>
              - <xs:sequence>
                  <xs:element name="name" type="xs:string" minOccurs="0" maxOccurs="1" />
                - <xs:element name="questions" maxOccurs="1">
                  - <xs:complexType>
                    - <xs:sequence>
                      - <xs:element name="question" maxOccurs="50">
                        - <xs:complexType>
                          - <xs:sequence>
                              <xs:element name="question_id" type="xs:positiveInteger" />
                              <xs:element name="category" type="xs:positiveInteger" />
                              <xs:element name="text" type="xs:string" />
                              <xs:element name="answered" type="xs:boolean" default="false" />
                              <xs:element name="multiplechoice" type="xs:boolean" default="false"
                                />
                            - <xs:element name="possible_answers">
                              - <xs:complexType>
                                - <xs:sequence>
                                    <xs:element name="answer" minOccurs="1" maxOccurs="8"
                                      />
                                  </xs:sequence>
                                </xs:complexType>
                              </xs:element>
                            </xs:sequence>
                          </xs:complexType>
                        </xs:element>
                      </xs:sequence>
                    </xs:complexType>
                  </xs:element>
                </xs:sequence>
              </xs:complexType>
            </xs:element>
          </xs:sequence>
        </xs:complexType>
      </xs:element>
    </xs:sequence>
  </xs:complexType>
</xs:element>
</xs:schema>
```
